# A Bayesian model for chronic pain

**DOI:** 10.3389/fpain.2022.966034

**Published:** 2022-09-16

**Authors:** Anna-Lena Eckert, Kathrin Pabst, Dominik M. Endres

**Affiliations:** Theoretical Cognitive Science Group, Department of Psychology, Philipps-University Marburg, Marburg, Germany

**Keywords:** chronic pain, interoception, graphical models, computational psychiatry, Bayesian inference, belief-propagation, predictive coding, free energy

## Abstract

The perceiving mind constructs our coherent and embodied experience of the world from noisy, ambiguous and multi-modal sensory information. In this paper, we adopt the perspective that the experience of pain may similarly be the result of a probabilistic, inferential process. Prior beliefs about pain, learned from past experiences, are combined with incoming sensory information in a Bayesian manner to give rise to pain perception. Chronic pain emerges when prior beliefs and likelihoods are biased towards inferring pain from a wide range of sensory data that would otherwise be perceived as harmless. We present a computational model of interoceptive inference and pain experience. It is based on a Bayesian graphical network which comprises a hidden layer, representing the inferred pain state; and an observable layer, representing current sensory information. Within the hidden layer, pain states are inferred from a combination of priors p(pain), transition probabilities between hidden states $p({\text{pain}}_{t+1}|{\text{pain}}_{t})$ and likelihoods of certain observations p(sensation∣pain). Using variational inference and free-energy minimization, the model is able to learn from observations over time. By systematically manipulating parameter settings, we demonstrate that the model is capable of reproducing key features of both healthy- and chronic pain experience. Drawing on mathematical concepts, we finally simulate treatment resistant chronic pain and discuss mathematically informed treatment options.

## 1. Introduction

Pain has an undeniable function in daily life. By signalling potential or actual bodily damage and directing both attention and behavior, it ensures the individual’s physical integrity in the long run (1). When touching a hot plate, for example, the individual will withdraw their hand immediately to avoid further tissue damage, and seek to soothe the acquired damage.

The same can not be said about chronic pain, where pain persists beyond the presence of acute injury (1). With around 20% of adults and children reporting symptoms of chronic pain, it is a highly prevalent disorder (2, 3). Chronic pain leads to significant losses of quality of life (adults: (4), adolescents: (5)). The disorder is associated with decreased levels of psycho-social functioning and productivity (6) and frequent utilization of healthcare services (7), causing a substantial economic burden (8–10).

Despite the ever-rising prevalence of chronic pain (11, 12), its underlying mechanisms remain poorly understood. Computational-level mathematical models (cmp. (13)) can help to elucidate the problem that the mind is trying to solve (e.g., detecting harm to the body) and where such solution attempts go wrong. Recent developments in cognitive computational neuroscience and machine learning may offer new opportunities for studying chronic pain from this statistical viewpoint.

A growing body of work suggests interoception, just like perception, follows probabilistic, Bayesian principles (14–18). Perceiving both the external world and the internal milieu requires effectively dealing with noisy, ambiguous and incomplete sensory information, usually from a multitude of sources and modalities. For this, the mind appears to rely on prior knowledge in the shape of a generative model of the environment, which is integrated with the current sensory input (19–21). Following this account, perception is not only shaped by the sensory information reaching the mind or body, but also by the predictions of the generative model this information is met with. This notion is summarized under the free-energy principle (22, 23).

The modulatory effects of prior knowledge on pain perception have been widely demonstrated - from the level of neural processing in the brain stem (24) through to self-reported pain intensity (25–28). For example, Tabor et al. (16) show that pain perception varies in dependence of an exteroceptive cue. When paired with a blue light (signalling safety), a noxious stimulus is perceived as less painful compared to when it is paired with a red light (signalling danger and heat). Further, placebo analgesia describes the phenomenon of experiencing pain relief after taking a placebo pill (15, 29, 30). In line with statistical accounts of pain, this further suggests an integration of expectations, or prior beliefs, with the physical sensory signal. In this context, Hechler and colleagues (2016) have instantiated Bayes’ theorem with pain and nociception-related variables to illustrate statistical computations underlying pain perception:

(1) $$p(\text{pain}|\text{sensation})=\frac{\;p(\text{sensation}|\text{pain})\;*\;p(\text{pain})}{p(\text{sensation})}$$

where p(pain∣sensation) is the **posterior** probability. The posterior determines the individual’s subjective experience of pain. It is proportional to the **likelihood** p(sensation∣pain) times **prior** probability of pain, p(pain). p(sensation∣pain) describes the probability of being exposed to a certain sensation (i.e., nociceptive signals) when the body or a specific body part is in a state of pain. Following this account, chronic pain has been described in terms of aberrant Bayesian inference (14). For example, averse life events may lead to a heightened prior expectation of pain, p(pain). This may underlie lowered pain thresholds and hyperalgesia frequently observed in chronic pain patients (31). Long-term learning experiences may alter the associations between sensory information and the inferred experience in patients. In other words, the likelihood term p(sensation∣pain) may be subject to learned biases in patients so that a broader range of stimuli is associated with pain compared to healthy controls (14). This may underlie allodynia, where harmless stimuli are perceived as painful.

We build on these previous lines of work and propose a quantitative Bayesian framework, implementing the above-mentioned considerations within a hierarchical and sequential model. It is built on the assumptions that (i) pain perception emerges from Bayes-optimal combination of sensory input and prior beliefs, (ii) chronic pain is characterized by maladaptive learning processes on longer time-scales, (iii) these learning processes result in generalizing, heightened expectations of pain p(pain) as well as (iv) heightened p(sensation∣pain), i.e. patients erroneously infer being in pain as the most likely cause of a wide range of sensations usually considered harmless.

Our formal implementation is based on the assumption that interoception is an inference problem that requires optimizing the generative models, i.e. the mental representation of causal relations between body state (i.e. a pain-free or pain state) and sensory (e.g. harmless or noxious) inputs. Using concepts from machine learning, such as variational inference, the system’s state is approximated (32, 20). Pain is represented in latent state variables and must be inferred from previous model states and sensory information, represented as observable variables. By representing perception and sensation on different computational levels, our model allows the formalization of non-veridical relationships between physical stimulus and pain perception. In other words, pain needs to be inferred using all available information, such as nociceptor firing rates (sensations), general pain expectations and specific pain expectations based on recent percepts. The model incorporates learning, which is implemented using message passing and the minimization of free energy over time (22, 33). We first describe the model’s architecture and features, before simulating the effects of the parameters. Here, we focus on the effects of a prior probability of pain, p(pain), a likelihood model p(sensation∣pain), and $p({\text{pain}}_{t+1}|{\text{pain}}_{t})$, which describes the probability of remaining in a state of pain between subsequent time steps t and t+1. To illustrate our results, chronic pain can emerge from a high and precise prior belief to be in pain p(pain), which is generally more likely than not being in pain (here over-lined, representing the complement of pain; $p(\bar{\text{pain}})$); $p(\text{pain})>p(\bar{\text{pain}})$, in patients. The prior is sharpened further with every time-step where pain is inferred as the most likely cause of a given sensation, leading to a stabilization of the system within this pathological state. The system then is resistant to correctly interpreting harmless sensory information, as represented in an ambiguous p(sensation∣pain).

## 2. Materials and methods

### 2.1. Model preliminaries

#### 2.1.1. Sequential data

Chronic pain develops over time, therefore an appropriate computational model has to be sequential in nature. Further, the state of the body a short moment ago is usually the best predictor for its current state. The brain seems to leverage this predictability to process sensory information more efficiently (22, 32). It is hence plausible to introduce a dependency of states over time into the model. Since the mind is assumed to constantly predict future sensory input, the model needs to accommodate longer time-series. The more recent past is usually more relevant to the current situation than the distant past. For example, what you saw three seconds ago is more relevant to your current situation than things you saw 5 weeks ago. The future state of a Markov chain is conditionally independent of all previous states, given the current state (Markov property). A Markov chain model fulfills the requirements of accounting for time-series dependencies while maintaining computational tractability and hence biological plausibility (for more details, see Appendix A).

#### 2.2.2. Perceptual hierarchies

**Figure 1:**
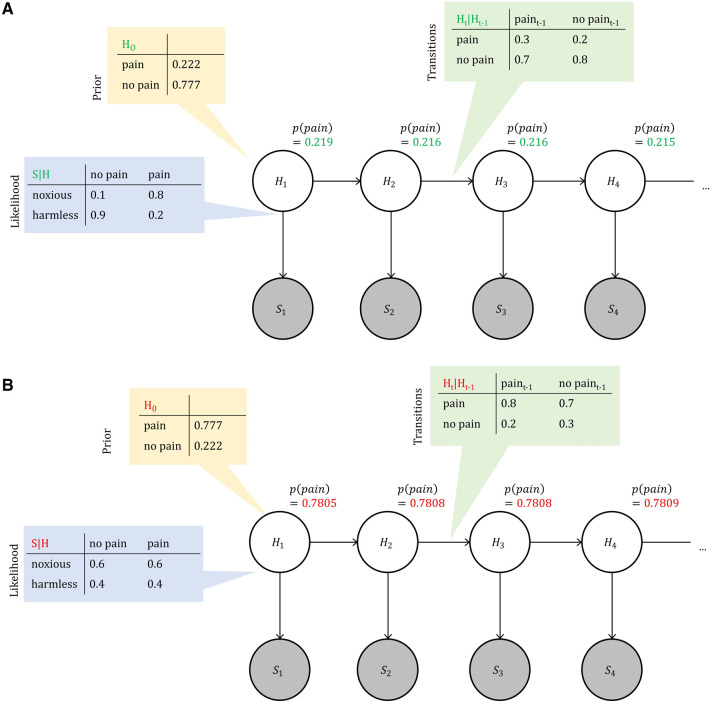
Bayesian network representation of pain perception. We model perception in time as a hidden Markov model, where unobservable (hidden) nodes $H_{t}$ form a Markov chain. These hidden nodes represent the state of the body, that the perceiving agent has no direct access to. Each hidden node connects to an observable node $S_{t}$, which represents a sensory (e.g. noxious) input. The connections indicate the direction of causation: body states cause noxious inputs in a healthy agent. Tables show exemplary settings of relevant probabilities for both healthy and chronic pain perception, where prior probabilities are chosen so that expectations are stable in time (see Appendix B for details).

While a given intensity of noxious stimulation induces intense pain in one subject, another one may barely experience discomfort (34). The non-veridical relationship between sensation and pain prompts the need for a differential representation of the two within a model. This large inter-individual heterogeneity in pain experience further suggests a crucial role of an individual’s generative model of pain experience. We use a Hidden Markov model (35, 36) with latent (hidden) variables, representing the pain state, or H, and observable variables, representing sensory input, or S; see Figure 1 for a graphical representation. Pain is represented in hidden state variables and functions as an indicator of the body’s integrity. This information is not directly accessible to the individual and hence has to be inferred from the available sensory information and prior expectations. From a Bayesian perspective, the experience of pain is determined by performing inference on hidden states, given the individual’s prior beliefs. Given the non-linear and inter-individually heterogeneous relationships between sensation (i.e. nociceptor firing after physical stimulation) and percept (pain experience), an appropriate model allows the distinction of the two. In our model, this is achieved via separate hierarchical layers (observable and hidden). Of note, this computational-level model (in the sense of (13)) does not translate directly to implementational, neuroanatomical levels relevant to pain perception (37).

#### 2.3.3. Inference and learning

The perceiving individual does not have direct access to their surroundings. It has to rely on information from its sensory organs, such as signals from sensory receptors, to obtain a representation of its surroundings. The accuracy of this information is crucial to the individual’s survival in a given environment. Since the information arriving at the sensory organs is noisy and ambiguous, the mind needs to infer the real environmental causes for the sensation. Both prior beliefs and sensory information appear to be represented in a probabilistic manner (38). Perception then is the Bayes-optimal combination of prior beliefs and said sensory information. Conscious experience is determined by the hidden state that is assigned the highest posterior probability. In the context of pain perception, inference means determining the extent to which an observation S=s allows the perceiver to update their knowledge about a hidden state H=h. For this purpose, it is necessary to infer the marginal probabilities of the latent variables from observations. We use the sum-product algorithm, also known as belief-propagation, as an efficient means of deriving marginal probabilities in singly-connected graphical models (36, 39). Inference and learning over time is governed by the imperative to minimize free energy, a principle which has been proposed as a general theory of brain function (22, 20, 40). Variational message passing, as proposed in this framework, has the advantage of both computational tractability and biological plausibility (22). The interested reader is referred to Appendix A for a detailed description of the approach to inference applied here. While inference in this framework refers to short-term conclusions about marginal probabilities of pain, learning refers to longer-term updates of model parameters. We here implemented batch-updates, where model parameters are updated once after the observation of a full time series. Psychologically, this would correspond to retrospective memory consolidation, for example during sleep.

## 3. Simulations

All models and simulations were implemented in Python (version 3.9.7), in particular the numpy package (version 1.21.2, (41)) and a package for variational inference with exponential family models by author D.E. (33). All plots were created using the matplotlib.pyplot library for Python (version 3.4.3, (42)).

A Hidden Markov Model with N=20 time-steps and free-energy learning over time is implemented (see Appendix A for details). Its hidden state variables, or nodes, $H_{t}$, represent the system’s internal state at time point t. There are two possible states in each state, ($H\in \{\mathrm{pain},\bar{\mathrm{pain}}\}$) which are inferred from the combination of previous nodes’ states and incoming sensory information. Sensory information is represented within the observable nodes $S_{t}$. There are two possible observations: noxious or harmless sensory information, or S∈{noxious,harmless}. Observable nodes can remain unobserved. The top-down factor contains the likelihood $p(S_{t}|H_{t})$ which allows explicit modelling of the association between sensory information and internal model state. The hidden variable nodes form a Markov chain, connected through additional free-energy factor nodes necessary for message passing. Here, the transition probabilities $p(H_{t}|H_{t-1})$ represent the implicit expectations of pain, or the pain prior, over time. In other words, transition probabilities translate into the individual’s expectation regarding the persistence of pain. A fixed and precise transition probability can constrain the model to arrive at a steady state, where conflicting sensory information does not alter the model’s inferred hidden state, which we will discuss in detail below. We systematically vary the above-mentioned parameters to capture healthy pain perception as well as one that is biased towards chronically inferring pain. While the underlying inference engine is the same in both cases, there are four starting points from which to manipulate the model’s behavior, also summarized in Figure 1.

1. The **prior** of the first hidden variable $H_{1}$;
2. The **likelihood function**, or the top-down factor connecting observable and hidden nodes, $p(S_{t}|H_{t})$;
3. The **transition probabilities** $p(H_{t}|H_{t+1})$ between the hidden variables;
4. The **sensory information** at observable nodes $S_{t}$.

Parameters are characterized by both the probabilities for specific states or observations, represented by their sufficient statistics λ; and their precision, formalized by their pseudocounts ν (see Appendix A for details). ν is referred to as the *pseudocount* as it keeps count of the number of observed data points.

*Healthy interoceptive inference* (“healthy observer,” from here onward) is characterized by a low, but precise prior expectation of pain (p(pain)=0.2,ν=100) and a precise likelihood function that allows accurate inferences based on incoming sensory information (e.g., $p({\text{noxious}}_{t}|{\text{pain}}_{t})=0.8,$ ν=100). Transitions towards pain-free states are more likely than transitions to painful states, as implemented in the transition probabilities between hidden states (e.g. $p(H_{t}=\bar{\text{pain}}|H_{t-1}=pain)=0.7$).

In contrast, *chronic pain inference* (“chronic pain observer”) is characterized by an increased prior expectation of pain (p(pain)=0.9,ν=100). The likelihood functions are imprecise and do not allow accurate inference from incoming sensory data, with $p({\text{noxious}}_{t}|{\text{pain}}_{t})=0.6$, ν=20. Transitions between hidden states are biased so that transitions into painful states are more likely than transitions into pain-free states, e.g. $p(H_{t}=\text{pain}|H_{t-1}=\bar{\text{pain}})=0.7$.

### 3.1. Mixed observations

**Figure 2:**
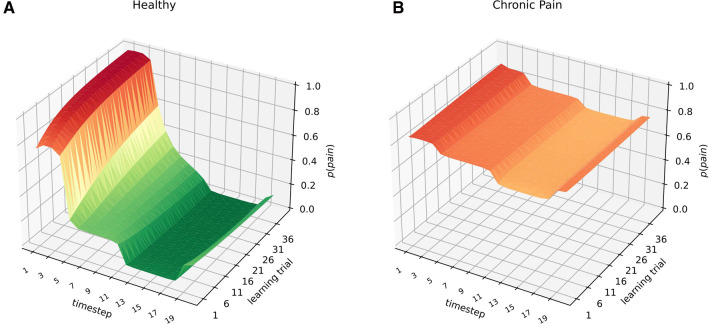
Healthy (**A**) and chronified pain inference (**B**). The X-axis shows the time-step, referring to e.g. times during a day. The learning trials on the Z-axis refer to moments of parameter updates, e.g. consecutive days, or longer-term memory consolidation and learning. On the Y-axis, the inferred marginal probabilities of pain are represented. Both models were exposed to *noxious sensory information* at nodes 1–5, and to *harmless sensory information* at nodes 12–18 for 40 learning trials. The inferred marginal probabilities develop dynamically in the case of healthy inference (left), whereas there are hardly any deviations from the prior beliefs in the case of chronified pain inference (right).

In a first simulation, both observers were exposed to the same sensory information: noxious input for time steps $S_{1}-S_{5}$, and harmless input st $S_{12}-S_{18}$ for 40 learning trials. Results are shown in Figure 2. In the healthy observer (Figure 2A), the exposure to noxious stimuli between time steps 1 and 5 leads to an acute and highly precise inference to be in pain. In contrast, the presentation of harmless sensory information (time steps 12–18) decreases the probability of inferring to be in pain drastically. In the chronic pain model, however, the response to acute noxious sensations follows a different pattern (Figure 2B)—the baseline probability of inferring pain is at ceiling levels already. In contrast, harmless information barely has any significant effects on the probability of inferring pain, which remains high.

### 3.2. Prolonged exposure

**Figure 3:**
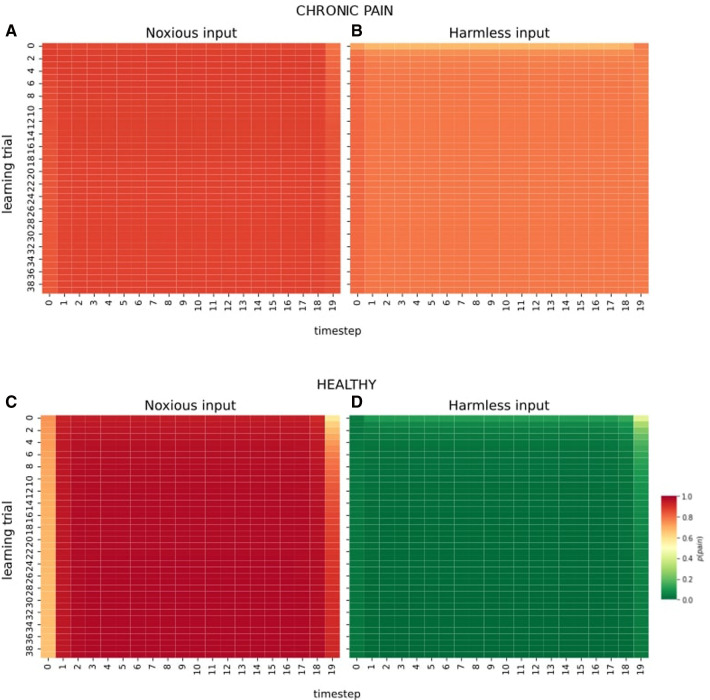
Effect of prolonged exposure to one type of information. The X-axis in all plots shows the time step (e.g., time steps during a day), and the Y-axis shows learning trials (e.g., memory consolidation at the end of each day). (**A**) and (**B**) show the observer with chronic pain exposed to 20 time steps and 40 trials of noxious information (**A**), and 20 time steps and 40 trials of harmless observations (**B**). (**C**) and (**D**) illustrate the same observational scheme under healthy pain inference. Of note, the marginal probabilities inferred at the first- and last time step differ from the probabilities inferred in intermittent nodes for two reasons. First, due to our model architecture, the first- and final nodes only receive messages related to sensory inputs from one neighbour, while intermittent nodes receive richer sensory information from their two respective neighbours (e.g., past and future time steps), leading to increased certainty about the hidden state. Secondly, we perform batch updates at the end of one time series. In psychological terms, this corresponds to retrospective memory consolidation during sleep. With increased learning trials (Y-axis), however, the marginal probability of inferring pain under noxious stimulation (panels **A** and **C**) increases as a consequence of learning.

In another simulation, the two observers are exposed to only-noxious vs. only-harmless sensory observations for all 20 time steps and 40 learning trials. Results are shown in Figure 3. In individuals with chronic pain (3A,B), the type of sensory information the system is exposed to does not result in significant changes of p(pain). Especially compared to the healthy inference model (3C,D), exposure to harmless information does not lead to decreased probabilities of inferring pain. In healthy inference, prolonged exposure to noxious information leads to acute heightened probabilities of experiencing pain. In contrast, harmless stimulation results in very low probabilities of inferring pain. Of note, the inferred probabilities of pain at the first- and final time step differ from intermediate time steps. This is an artifact caused by the model architecture with a definite end node, and our batch-updates (learning) that are performed at the end of one time series (see figure caption for details).

## 4. Treatment-resistant chronic pain: a null space problem?

Some forms of chronic pain are remarkably resistant to psychotherapeutic (43) or pharmacological (44) intervention, which has led to calls for ever more radical treatments such as transcranial magnetic stimulation, or TMS, (45), electroconvulsive therapy (46) or ketamine infusions (47).

A computational model of chronic pain therefore needs to be able to demonstrate a rigid, “treatment-resistant” pain experience over time. In the present model, this translates to a high temporal stability of the inferred, latent pain-state, regardless of the varying sensory input at the observable nodes (i.e., psychotherapy) or modulation of the sensations (i.e. pharmacotherapy). To derive the parameter settings necessary to arrive in a fully stable model state, we borrow the concept of the null space from linear algebra (36).

The null space of a matrix A contains all vectors $\vec{x}$ such that $A\vec{x}=0$ (48). We are interested in the null space of the matrix that constrains the transition probability distributions $p(H_{t+1}|H_{t})$ to yield a $p(H_{t}=\mathrm{pain})$ after waiting for a sufficiently long time, i.e. we are looking for the stable states of the Markov chain. In other words, we are interested in changes to the transition probabilities that have no effect in the long run – i.e. a resistance of the system to treatment.

**Figure 4:**
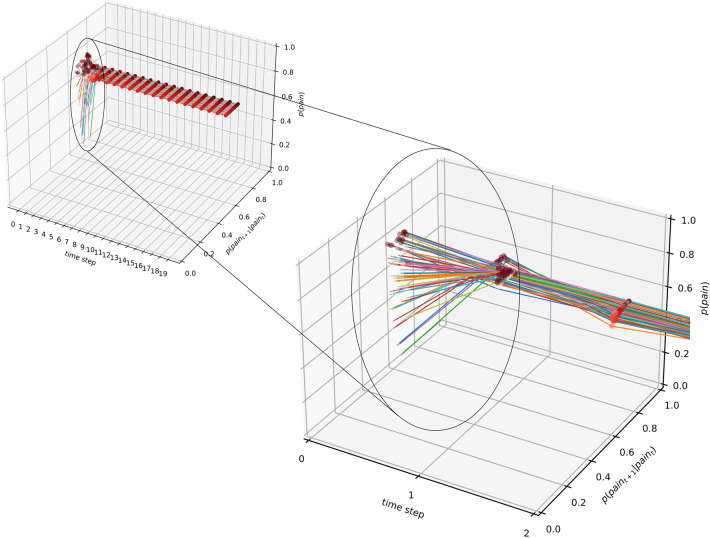
Null space of deviations from final state: over time, the marginal probability of inferring pain approaches and stabilizes within ranges of the preset marginal, here, p(pain)=0.7. Random changes to the prior expectation of pain (at time step 0, illustrated by the different-colour lines) are overridden within two time steps, and the marginal probability approaches the predefined value.

We demonstrate in Appendix B that for each marginal p(pain), there are transition probabilities $p(H_{t+1}|H_{t})$ that satisfy this condition. Resulting is a line, the one-dimensional null space, containing matching transition probabilities for a given marginal probability of pain. The combination of marginal and derived transition probabilities has a marked effect on the inferred state: it remains stable within the range of the preset marginal probability of pain, irrespective of the quality of observations made, or changes to the prior. In Figure 4, we illustrate the example of the marginal p(pain)=0.7. First, as above, a model with 20 time steps and 40 learning trials is created. The prior $p(H_{0}=\text{pain})$ on the first time step is chosen randomly. Crucially, we sample transition probabilities from the null space and then sample the inferred probability of pain at the last time step. It becomes evident that within a very short period, the inferred probability of pain stabilizes at the pre-defined marginal p(pain) of 0.7—regardless of any changes to the prior information.

### 4.1. Therapy for chronic pain

**Figure 5:**
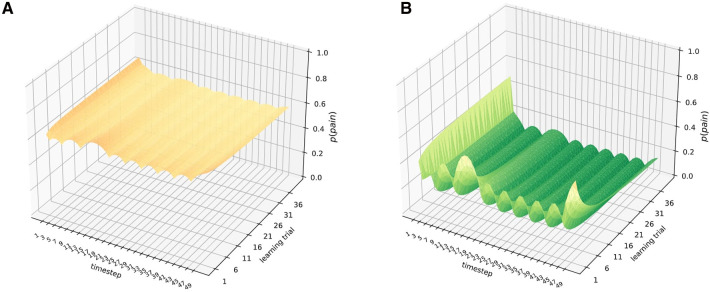
Simulation of ten sessions of exposure therapy in the nullspace of marginal p(pain)=0.7, with ambiguous and imprecise likelihoods (**A**) and precise and accurate likelihoods (**B**). On the X-axis are the time steps, on the Y-axis the marginal probabilities of pain, and on the Z-axis the learning trials per time step. The combination of null space derived transition probabilities and an imprecise and ambiguous likelihood model (p(noxious∣pain)=0.6, p(harmless∣pain)=0.4, $p(\text{noxious}|\bar{\text{pain}})=0.6$, $p(\text{harmless}|\bar{\text{pain}})=0.4$) renders the repeated presentation of innocuous sensory information rather inefficient: inferred probabilities of pain remain within the range of the predefined marginal. When the likelihood model is precise and unambiguous (p(noxious∣pain)=0.8, p(harmless∣pain)=0.2, $p(\text{noxious}|\bar{\text{pain}})=0.1$, $p(\text{harmless}|\bar{\text{pain}})=0.9$), however, the presentation of harmless sensory information is more efficient in reducing the inferred probability of pain.

Exposure therapy is an effective treatment for chronic pain, where patients with high levels of fear-avoidance are gradually exposed to stimuli or movements that elicit fear (49). Relating the idea of exposure therapy to our model, its goal would translate to changing the interpretation of harmless sensory information so that the inference becomes less biased towards pain. As an example, a person with chronic low back pain may avoid lifting a basket with groceries out of their car. In graded exposure therapy, the therapist would guide the patient through lifting baskets with increasing weight, all while closely monitoring the patient’s fearful assumptions and physical sensations. Over the course of the exposure treatment, we would expect a change in p(lift-basket∣pain) in this patient. Altered likelihoods may lead to less guarding behavior in patients, an increase in corrective sensory information and, ultimately, remission. In the specific case of fixed transition probabilities, however, this approach may not be sufficient. This picture would be in line with treatment-resistant chronic pain. The maintenance of p(pain) in these patients is mostly determined by the transition probabilities, while incoming sensory information is mostly disregarded. We simulated two observers who receive ten sessions of exposure therapy (presentation of harmless sensory information for one time step) over the course of fifty time steps. In both cases, the pre-set marginal probability of inferring pain is p(pain)=0.7, and the transition probabilities were sampled from the respective null space of a marginal p(pain)=0.7. Results are illustrated in Figure 5. In the case of the first patient, the likelihood functions, p(sensation∣pain) were ambiguous (p(noxious∣pain)=0.6, p(harmless∣pain)=0.4,p(noxious∣ $\bar{\text{pain}})=0.6$ $p(\text{harmless}|\bar{\text{pain}})=0.4,$ and imprecise (ν=20). In the simulation, the marginal probability of inferring pain dips slightly, before moving towards the initial, high p(pain)=0.7 again. In a second case, the likelihood functions were intact, that is, accurate (*p*(noxious | pain) = 0.8, *p* (harmless | pain) = 0.2, *p*(noxious $\bar{\mathrm{pain}})=0.1,p(\text{harmless}|\bar{\text{pain}})=0.9$) and precise (ν=100). Here, the inference moves away from the high prior faster and remains relatively pain-free for longer.

## 5. Discussion

### 5.1. A Bayesian model for chronic pain

The experience of pain seems to be the result of an inferential process, where prior expectations are integrated with current sensory information over time (14, 16). This process is probabilistic and non-linear, with large inter-individual differences in the relationship between physical stimulation and pain experience. While statistical accounts of pain have gained momentum over the past decade, insights into computational underpinnings of the chronification of pain have been sparse (50, 18, 14).

We propose a Bayesian model for interoceptive perception and pain experience in chronic pain. Drawing on machine learning concepts such as belief-propagation and free-energy minimization, we demonstrate that the model captures well-studied phenomena such as decreased pain thresholds and allodynia (i.e. perceiving innocuous stimuli as painful) in individuals with chronic pain.

Our “chronic pain” observer is characterized by heightened prior expectations of pain, aberrant associations between sensory input and pain perception and heightened assumptions about the persistence of pain. In our model, this refers to overly precise priors for being in a state of pain, p(pain)), ambiguous top-down or likelihood functions p(sensation∣pain) and more rigid transition probabilities between hidden states $p({\text{pain}}_{t}|{\text{pain}}_{t-1})$, respectively.

### 5.2. Chronic pain as biased inference

It has been suggested that chronic pain is associated with a broad and heightened expectation of pain (51, 14, 24). Heightened expectations of pain may stem from early averse life events or injuries (52), or social factors such as an overprotective, fearful parental style (14). In the patient model, a heightened pain prediction led to inferring to be in pain more readily compared to the healthy model. Conflicting innocuous sensory stimulation hardly influenced the inferred state. In accordance with this, empirical studies suggest chronic pain patients may not attend to sensory information but rather rely on their prior expectations of pain (53, 54). Our observations fit into a larger discussion about expectations as core features of a multitude of mental disorders (51, 14). A further aspect of our model is that harmless sensory information is associated with pain over time. This phenomenon is commonly observed in patients and referred to as allodynia (55, 14). The Bayesian Brain hypothesis postulates that the perceiving individual perpetually tries to infer the real-world causes for incoming sensory information (22). We here present a perspective on pain as an abstract percept of the body’s state. Following this view, pain is an inference on a real-world “cause” (cmp. (56)), bodily damage, based on all available prior- and current sensory information. The chronic pain patient infers pain from a wide range of sensory stimuli that would not be perceived as painful by healthy controls. Further, the associations between sensory information and hidden state are noisy and unclear in the chronic pain observer. While a healthy observer maintains precise beliefs about the external causes of harmless sensory input, p(harmless∣pain), this probability is completely ambiguous in our patient model. Over time, the chronic pain patient is less and less likely to infer a pain-free state from any type of incoming sensation.

Contrarily, a flexible and highly dynamic pattern can be observed under healthy interoceptive inference. Here, noxious stimuli are associated with pain, and a pronounced and acute state of pain is inferred from incoming harmful stimuli. However, once the stream of harmful sensory input stops, the probability of pain normalizes very quickly. This dynamic pattern is in line with the function of pain as a warning signal—motivating quick and adaptive behavior change following a threat to the individual’s physical integrity (57).

Note that our results also hold under the active inference framework (23, 58). Canonical versions of active inference contain a generative model that is very similar to ours (Hidden Markov model, HMM), with the difference of an additional layer that models an agent’s actions, or choices (59). Actions then influence the transitions between states so that more likely, or expected states under the current observations, are reached. In other words, in active inference, an agent infers the hidden state from the combination of prior beliefs, sensory information and their own actions. As an example, consider the case of a child with chronic abdominal pain who involuntarily keeps picking at her abdomen (checking behavior, example from (14)), so that increased pain is experienced. Under active inference, this can be interpreted as causing observations (noxious sensations) that are expected under the most likely state (pain). Acting directly towards bringing about a certain observation changes the causal relationship between observation and hidden state (i.e., “do-operations”, (60)). However, it is impossible to act directly on one’s nociceptors, so that pain still needs to be inferred from sensations, even when the noxious stimulation is self-induced.

### 5.3. Exposure and the null space of psychotherapy

If the individual performs implicit Bayesian inference over hidden states, then we here demonstrate that this inference can be biased towards inferring pain. This has important implications for the treatment of chronic pain patients. Cognitive behavioral therapy (CBT) shows statistically significant, but small effects on certain aspects of chronic pain (pain, mood, disability, pain catastrophizing; (61)). Techniques usually include relaxation training, behavioral activation (i.e. engaging in behaviors that were previously avoided due to fear of pain), setting behavioral goals, problem-solving training and cognitive re-structuring (61, 62). Further, exposure therapy has shown promising results in the treatment of chronic pain (63, 64, 49). Exposure therapy seems to reduce disability more effectively than CBT in patients with chronic lower back pain (49). It is designed to specifically target emotional responses to pain- or expectations of pain, which lead to excessive guarding behaviors. Guarding behaviors, in turn, may limit the opportunity for receiving corrective, conflicting sensory information, leading to the solidification of fearful pain beliefs (65, 66). However, we show that inference can stabilize towards pain, even in light of conflicting sensory information. Specifically, when the transition probabilities between hidden states have a stable state with high marginal pain probability, a simulated patient model returns to the inference of being in pain while harmless sensory information is provided. In other words, the initial deviation from the high pain state, which might have been reached by therapeutic means, has vanished. This simulation could be a model for treatment-resistant chronic pain. We further show that these patients may be best served by targeting their beliefs about the associations between sensory stimuli and pain expectations (likelihoods). When likelihoods allow the precise and accurate association between sensory stimuli and pain, exposure to harmless sensory information leads to more promising results. However, when the patient does not have accurate likelihoods, exposure to certain sensory stimuli may not be a promising approach. It can hence be derived that patients may need to re-learn precise and accurate inferences about different types of sensory stimuli, ideally before being guided through exposure therapy.

**Figure 6:**
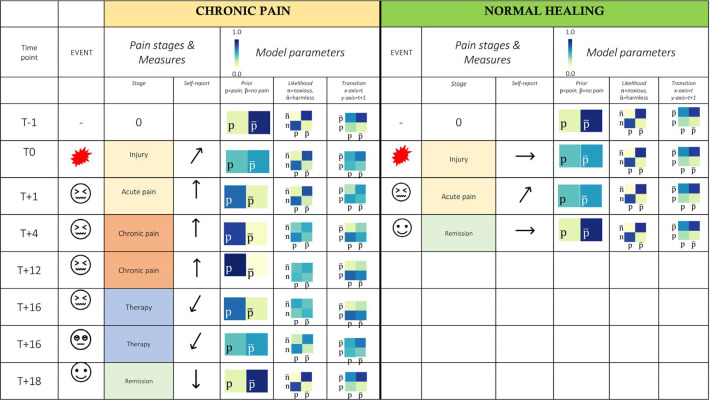
Tabular overview of patient experience and model predictions. *Timepoints*: T ± month, *Model parameters*: expected pattern of change in the model parameters underlying chronic vs. acute pain experience; where p, pain; $\bar{\;p}$, pain-free state; n, noxious input; $\bar{n}$, harmless input. *Self-report*: Expected values, ↑, increased values compared to baseline; ↓, decreasing values; →, average values; *, there is some evidence that the generative model of patients with chronic pain is biased towards pain from childhood on, e.g. via traumatic experiences, abuse and earlier chronic pain experiences (68–70).

One can speculate about the role of pharmacotherapy in the context of our model. Potentially, analgesics target the patient’s likelihood model by inhibiting the transmission of noxious information from pain receptors (67). This assumption requires empirical testing with appropriate data of medicated chronic pain patients. In this case, combining pharmacological and psycho-therapeutical strategies may be promising. More data is necessary to assess which treatments target what model parameter. For an overview of expected self-report scores and hypothesized underlying model parameters, see Figure 6.

In Figure 6, we illustrate two hypothetical patients, one developing chronic pain after an acute injury, whereas the other patient does not develop chronic pain beyond acute injury.

In the chronic pain patient, pain persists for prolonged periods post injury. Empirically, we expect to see pain and disability scores that are persistently heightened in the months following injury. In experiments (i.e. fear conditioning, pain ratings following noxious stimuli), our model predicts increased levels of prior expectations of pain (note the shift in probabilities over time), as well as decreased precision of sensory information, corresponding to an ambiguous and imprecise likelihood model. Finally, for pain to persist in a very rigid way, transitions towards a pain-free state become more unlikely until therapy can reverse this effect. We can speculate that likelihood models may be treated via medication-supported exposure therapy, whereas prior beliefs could be best targeted using CBT.

In the acute pain patient, the injury is followed by a quick recovery and remission into a pain-free state. The noxious stimulus leads to a transient state of experiencing pain, including an elevated prior expectation of pain, which is a consequence of learning about an acutely noxious stimulus. Disability and pain experience as assessed with self-report measures are elevated in times of acute injury. With the injury healing and the noxious information subsiding, the pain-free state is re-established within 4 months. Note that the likelihood and transition models are assumed to stay relatively intact in this patient.

### 5.4. Expectations as primary therapy target

Following our model, there are three factors that could be promising targets for patients with chronic pain. Firstly, therapy could target the heightened and generalized expectations of pain. This is in line with Rief et al. (51), Panitz et al. (71), who discuss the importance of psychological interventions that target expectations. Secondly, our model suggests a need for re-learning the associations between sensory information and the state of pain. This could be achieved via gradual, guided exposure to harmless stimuli in therapy. Lastly, a patient’s belief about the persistence of pain over time could be targeted specifically with cognitive interventions (e.g. retrospective evaluations, re-learning to differentiate pain intensities), which may be especially relevant in cases of treatment-resistant chronic pain.

### 5.5. Limitations

The presented model makes several simplifying assumptions and can be extended in numerous ways. Currently, latent- and observable variables could only take one of two possible states for the sake of simplicity. Continuous random variables could allow for more complex conclusions. It is generally feasible to perform inference on graphical models with continuous random variable nodes (72, 73). However, this added complexity might detract from our main conclusions, which we expect to hold in the continuous case, too. In future work, we will extend our model to continuous sensory signals and internal states.

Further, future efforts to extend this framework should account for the vast heterogeneity that is found among chronic pain patients. Assuming a multitude of underlying etiological factors, they might need differential representation in quantitative terms as well.

With this model, we focus on computational and psychological aspects of pain inference. We have formalized a hypothesis about the generative process underlying chronic pain. While several salient features of acute and chronified pain perception are captured by our model, we do not have access to the true generative process and can hence not draw any definite conclusions about it.

Besides psychological factors (e.g. beliefs), a multitude of additional factors could be considered when extending this model. As Kiverstein and colleagues (2022) note, a model of chronic pain needs to take on a bio-psycho-social perspective, integrating a multitude of empirical findings under one approach. For example, pain experience seems to be alleviated by the presence of a close friend or partner, i.e. social support (74). In a recent review, Mohr and Fotopoulou (2018) argue that social support may serve as a security signal which renders prediction errors caused by threats to the physical integrity less precise. Future iterations of this model may be extended to incorporate these findings, e.g. by means of a context-layer. Here, however, we show that important characteristics of chronic pain experience can be simulated from a Bayesian perspective, by assuming that a) sensory information relates to pain experience through (biased) parameters of a generative model and b) the inferred states are marked by correlations, or stability, over time.

### 5.6. Future directions

Rigid expectations that are not sufficiently constrained by incoming sensory information seem to lie at the heart of several mental disorders. Psychosis, for instance, can be regarded as resulting from an exaggerated and inflexible reliance on prior expectations (75, 76). Computational accounts of mental disorders can provide novel mechanistic insights into their etiology, exacerbation and maintenance over time (77).

The computational model of chronic pain presented here can be applied to empirical data. Below, we outline possible avenues towards testing the model presented here.

Firstly, data from learning experiments in chronic pain patients and controls could shed light on the real-world significance of the model’s parameters. In fear conditioning experiments, participants learn the association between a painful unconditioned stimulus (UCS, such as e.g. heat stimulation with a thermode or electric shocks) and unrelated, conditioned stimuli (CS, such as a visual cue). In a first step, significant group differences in model parameters would need to be investigated. Then, given their heightened pain prediction, our model would predict decreased pain thresholds in patients with chronic pain. Biased likelihood terms may lead to altered learning about the UCS-CS contingencies in chronic pain patients. Similarly, other assumptions that were used to furnish this model could be tested empirically (e.g. biased and imprecise likelihood models, heightened prior predictions of pain, or rigid and exaggerated beliefs about the persistence of pain in chronic pain patients).

A key challenge to empirical tests of our model in humans is the need to approximate observable sensations, i.e., nociceptor firing. Sensations can be approximated by measuring physical properties of experimental stimuli, such as the temperature of a heat thermode, the current used for electric stimuli, or the intensity of mechanical pressure. To empirically test our model, future studies could assess the relationship between physical sensations and the pain percept. For this, concurrent measurements of sensations (or proxies thereof, such as physical stimulus properties) and reports on the pain percept (e.g. pain scores) are necessary. Pain scores in response to stimuli with different intensities could be collected from healthy individuals and chronic pain patients. We would expect to find significant group differences with respect to prior, transition- and likelihood model parameters. Such data could further shed light on an important detail of patient experience: what model parameter is most relevant to the chronification of pain and the experience of patients? In all cases, the full Bayesian model would need to be compared to both simpler models, such as e.g. single-trial vs. sequential models, and more complex models, such as deeper predictive hierarchies, in a model comparison.

### 5.7. Conclusion

A novel Bayesian model is able to reproduce common features of healthy- and chronified pain perception. Machine learning approaches, such as a hidden Markov model and variational inference, allowed the exploration of parameters that may underlie the development of chronic pain. We have demonstrated that this model is able to capture many phenomena observed in individuals with chronic pain, and during acute, healthy pain perception. Additionally, the model was able to simulate rigid, treatment resistant chronic pain. Further research could significantly advance our mechanistic understanding of chronic pain, which may help to inform treatment selection.

## Figures and Tables

**Figure A1:**
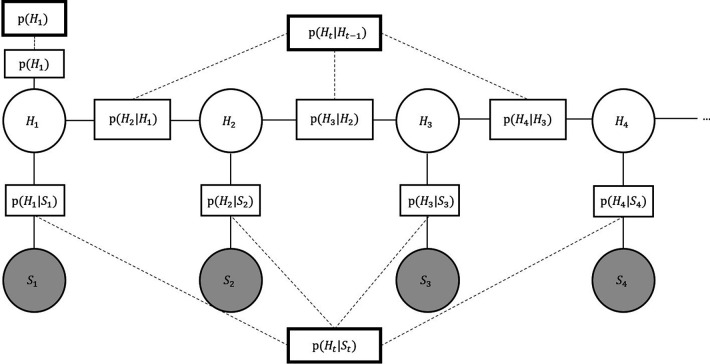
Graphical model (fragment). **Graphical model** depicting the inter-dependencies between hidden $H_{t}$ and observable $S_{t}$ variables. Connected to each variable node, factor nodes are necessary to perform variational inference message passing. This approach is computationally efficient because it allows message passing without re-instantiating the model at each time-step. The observable nodes $S_{t}$ represent incoming sensory information, S∈{noxious(1),harmless(0)}. The hidden states, representing the system’s internal model, can in turn take on the values 0 (no pain) or 1 (pain), or $H\in \{\text{pain},\bar{\text{pain}}\}$. The likelihood $p(S_{t}|H_{t})$ is the top-down factor in this model and allows quantifying the association between the model’s state $H_{t}$ and the observed sensory input at $S_{t}$. The hidden variable nodes form a Markov chain and contain transition probabilities $p(H_{t}|H_{t+1})$, which can be interpreted as the development of the pain prior over time. In individuals with chronic pain, we here assume that $p(H_{t}=\text{pain}|H_{t-1}=\text{pain})>p(H_{t}=\bar{\text{pain}}|H_{t-1}=\text{pain})$.

**Figure B1:**
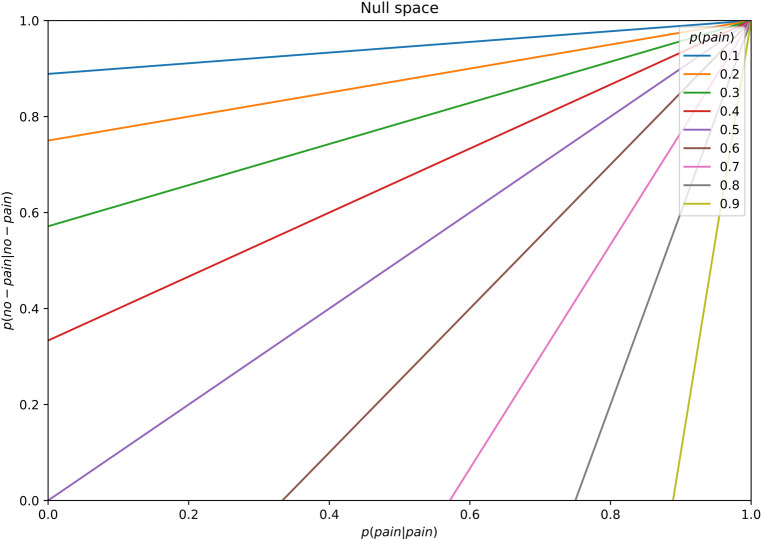
Illustration of null space of transition probability deviations for different marginal $p({\text{pain}}_{\infty })$. We show that when the transition probabilities $p({\text{pain}}_{t+1}|{\text{pain}}_{t})$ and $p({\bar{\text{pain}}}_{t+1}|{\bar{\text{pain}}}_{t})$ are sampled from the one-dimensional null space of a given marginal probability of pain $p({\text{pain}}_{\infty })$, the marginal probability $p({\text{pain}}_{t})$ will approach and stabilize within the range of $p({\text{pain}}_{\infty })$. Here, we illustrate the transition probabilities for $p({\text{pain}}_{\infty })\in [0.1,0.9]$ (legend).

## Data Availability

All code used in this manuscript is publicly available here: https://data.uni-marburg.de/handle/dataumr/163.

## Appendix A. Variational inference

Graphical models are a useful tool when representing and manipulating joint probability distributions. In the present context, we are interested in characterizing the mind’s probability computations of being in pain, given a series of noxious or harmless observations, i.e. *inference* (36). For this purpose, we use a modified state-space model architecture, i.e. a Hidden Markov Model (36). It features a chain of unobservable nodes, representing the inferred pain state at different time points. Each unobservable node is connected to one observable node representing sensations at the same time point (see Figure A1).

Assuming that hidden state inference evolves in an auto-correlated manner over time seems plausible because pain states tend to persist at least for some time. For this reason, a Markov chain of hidden states was selected as the basis of the model. The Markov property states that inference at a given future node, $S_{t+1}$, is conditionally independent (⊥) from all but the previous states $S_{t}$:

(A1) $$S_{t+1}⊥S_{t-1}|S_{t}$$

In other words, the future state $S_{t+1}$ does not depend on the distribution of the past state $S_{t-1}$ given the current state $S_{t}$.

It follows that the conditional distribution for the current state $S_{t}$ given *all* previous S is given by only its predecessor’s state $S_{t-1}$:

(A2) $$p(S_{t}|S_{1},\ldots ,S_{t-1})=p(S_{t}|S_{t-1})$$

The main inference goal is the estimation of the hidden random variables’ $H_{t}$ state, given a series of observations. This is expressed in the random variable’s respective marginal probabilities. Computing marginal probabilities is challenging, because it usually entails computing the sums of integrals over high-dimensional spaces. There are a number of widely used algorithms for efficient inference on graphical models. However, to perform inference with message-passing algorithms, we first need to transform the Bayesian network to a factor graph. A graphical representation of the factor graph is illustrated in Figure A1 (36). A factor graph represents the factorization of a function, or here, the probability distributions of interest. In other words, it provides a graphical representation of how probabilities of certain events depend on adjacent events in the observable and hidden layers. A factor graph consists of variable- and factor nodes, denoted by circles and squares, respectively. In our model, variable nodes can take on a binary set of values (here; $pain,\bar{pain}$ for the hidden nodes, and noxious,harmless input in the observable nodes). They can be observed or unobserved. Factor nodes contain exponential family distributions, parameterized by their natural parameters. These parameters represent probabilities for either transitioning between different hidden states or for observing certain types of sensory information, given a specific latent model state. We here further introduce hypernodes, denoted by thick squares, that govern the learning of factor-inherent natural parameters over time. All nodes are connected via edges to their respective neighbour nodes.

Once transformed, it is now appropriate to apply inference algorithms to the factorized graphical model. Sum-product message passing, also known as belief propagation, is a well-known algorithm that allows the efficient computation of marginal probability distributions on graphical networks without loops (39). In sum-product message passing, all nodes compute sums and integrals locally, before passing the results on towards the set of their neighbours in the shape of messages (72). In the present use-case, all variables are binary. In sum-product message-passing, the marginal probabilities for each state of a variable node V are given by the product of all incoming messages from factor nodes F:

(A3) $$p(V)=\prod_{l\in ne(V)}\mu_{F_{l}\to V}(V)$$

where ne(V) are the are the factor nodes’ neighbouring the variable node V, and conversely for factor nodes. $\mu_{F_{l}\to V}$ is the message received from factor node $F_{l}$. Variable nodes can only have factor nodes as neighbours. Messages from a factor node F to a variable node V are given by

(A4) $$\mu_{F\to V}(V)=\sum_{V_{1}}\cdots \sum_{V_{M}}F(V,V_{1},\ldots ,V_{M})\prod_{m\in ne(F)\setminus V}\mu_{V_{m}\to F}(V_{m})$$

Here, the product of the factor and all messages the factor node received from its neighbouring variables nodes except V, ne(F)∖V (a set of variable nodes without the recipient node), $\mu_{V_{m}\to F}(V_{m})$ is computed and summed over all variables except V. When ne(F)=None, that is, F’s only neighbour is V, the factor node sends its factor information:

(A5) $$\mu_{F\to V}(V)=F(V).$$

Messages from variable node V to a neighbouring factor node are given by

(A6) $$\mu_{V\to F}(V)=\prod_{l\in ne(V)\setminus F}\mu_{F_{l}\to V}$$

that is, V computes the product of all received messages from neighbouring factor nodes $\mu_{F_{l}\to V}$ except F. ne(V)∖F is empty when F is the only neighbour of V, in which case the message reduces to

(A7) $$\mu_{V\to F}(V)=1$$

where the value 1 is sent from unobserved nodes. Otherwise, the message value corresponds to the observation made at V. Note that variable nodes can also remain unobserved.

We here adapt this scheme and transform messages into log-space to avoid over- or underflow of the usual floating point computer arithmetic. With this, the marginal of a variable V is now computed via

(A8) $$p(V)=\exp\left(\sum_{l\in ne(V)}\log(\mu_{F_{l}\to V}(V))\right),$$

i.e., the marginal of a variable node is given by the sum of all incoming, log-transformed messages ($\log(\mu_{F_{l}\to V}(V$)) from factor nodes $F_{l}$.

Exact inference can become computationally inefficient or intractable in case of continuous variables. This is the case in our model due to the natural parameters of the probability distributions. To avoid this, we use variational inference and the lower bound approximation as implemented in the free-energy framework (78, 79, 36). Instead of estimating the (potentially intractable) posterior p(X∣D), with X representing model parameters and data D, an approximate distribution q(X∣D) is estimated. This further sidesteps the increasingly costly computation of sums over all variables and their values by introducing more simple update rules in order to compute the posterior distribution. The optimal q(X∣D) is estimated by maximizing the lower bound on the marginal log-likelihood of the data D, log⁡(p(D)), also referred to as the evidence lower bound (ELBO, (80)). In the present case, the marginal log-likelihood of D is given by

(A9) $$\log(p(D))=\log\left(\sum_{x}p(D,X)\right)=\log\left(\sum_{x}q(X)p(D|X)\frac{\;p(X)}{q(X)}\right)$$

Meaning that the marginal log-likelihood of the data is given by the sum over all joint probability distributions p(D,X), or the sum of an approximate distribution of X, q(X), and conditional probabilities of the data given X, p(D∣X) and a normalization factor p(X)/q(X). The last term of Equation A9 represents a factorization of log(p(D)).

When trying to approximate log⁡(p(D)), there are two essential ingredients: Jensen’s inequality and the Kullback-Leibler divergence (81, 82, 36). Applying Jensen’s inequality for convex functions, it follows that

(A10) $$\log\left(\sum_{x}q(X)P(D|X)\frac{\;p(X)}{q(X)}\right)\geq \sum_{x}q(X)\log\left(\;p(D|X)\frac{\;p(X)}{q(X)}\right).$$

The log sum of the factorized marginal probability distribution of p(D) is larger or equal to the sum of all approximate distributions q(X) times the log-normalized conditional probability distribution p(D∣X).

In a second step, we decompose the right-hand side of Equation A10, using log⁡(x∗y)=log⁡(x)+log⁡(y) into the expected likelihood and the Kullback-Leibler (KL) divergence between the approximate posterior and prior. The KL divergence can be interpreted as the dissimilarity of two functions. In this context, we are interested in the dissimilarity between our prior and the approximated marginal probability p(D).

(A11) $$\begin{array}{rl} \log & \left(\sum_{x}q(X)p(D|X)\frac{\;p(X)}{q(X)}\right)\\ & \geq \langle \log(p(D|X)\rangle_{q(X)}-KL(q(X)\|p(X)) \end{array}$$

where KL(q(X)‖p(X) is the non-negative KL divergence (81, 36) between prior p(X) and approximate distribution q(X). It follows that a lower-bound L(q(X),D) on the marginal log-likelihood log⁡(P(D)) is given by

(A12) $$L(q(X),D)=\langle \log(p(D|X)\rangle_{q(X)}-KL(q(X)\|p(X))\leq \log(p(D)).$$

Maximising the lower bound L w.r.t. q(X) is equivalent to optimising the approximation between p(X) and q(X). When these conditions are met, updated model parameters will improve the approximation of p(X) over time.

We now turn to describing the parameter updates in more detail. Variational inference schemes with identities vastly facilitate parameter updates by re-parameterizing the messages that are sent within the graphical model (36, 33). We specifically base parameter updates on exponential family distributions. The main reason for this choice is the mathematically convenient property of conjugate priors. A conjugate prior ensures that the prior probability distribution and the posterior probability distribution come from the same distribution family (36), which vastly facilitates parameter updates.

In the present model, we use the multinomial distribution, which in its standard form is given by (see (33))

(A13) $$P(x|q)=\prod_{k=1}^{K-1}q_{k}^{x_{k}}{\left(1-\sum_{k=1}^{K-1}q_{k}\right)}^{x_{K}}.$$

The multinomial distribution is a generalization of the Bernoulli distribution to K possible outcomes (36). In it, multinomial random variables x are represented by vectors with K components. Each component is either 0 or 1, $x_{k}\in \{0,1\}$ and all components sum up to 1, $\sum_{k=1}^{1}x_{k}=1$. $q=q_{1},\ldots ,q_{K}$ are the respective probabilities that $x_{k}=1$, i.e. we use a 1-out-of-K or one-hot representation.

The conjugate prior on the multinomial distribution is given by the Dirichlet distribution. The density of the Dirichlet distribution is given by Endres (33):

(A14) $$p(q|\alpha )=M\prod_{k=1}^{K-1}q_{k}^{\alpha_{k}-1}{\left(1-\sum_{k=1}^{K-1}q_{k}\right)}^{}\alpha_{K}-1.$$

where M is

(A15) $$M=\frac{\Gamma (\sum_{k=1}^{K}\alpha_{k})}{\prod_{k=1}^{K}\Gamma (\alpha_{k})},$$

and Γ(x) is the gamma function.

Variational inference allows for learning over time and avoids computationally costly new instantiations. For this, we need to introduce free energy nodes with natural parameters η (given by sufficient statistics λ and pseudocounts ν) into the graphical model. Free energy factors (in the following: factors/ factor nodes) are described by conjugate prior and exponential family distributions introduced above. Parameter updates in this context lead to improved model predictions so that here, we can interpret the updating process as learning over time.

Further efficiency is introduced into the model by incorporating hypernodes, e.g. $p(H_{t}|H_{t-1})$ and $p(S_{t}|H_{t})$ in Figure A1. Hypernodes ensure equal prior and posterior distributions of $P(H_{t}|H_{t-1})$ and $p(S_{t}|H_{t})$ at each time step t. They are connected to the more local, time point specific factor nodes implicitly via the lower bound, so that no loops occur and the application of belief-propagation algorithms remains feasible. In this design, each factor node’s parameters ν and λ are set to the specific parameter values of their associated hypernode. Hypernodes are not involved in sum-product message passing. Also, local factor parameters are not updated individually, but rather, the hypernodes’ parameters are updated after message-passing and then passed on to the local factor nodes. It can be shown that updates of hypernode parameters are done via:

(A16) $$\tilde{\nu }\;:\;=\nu +r$$

(A17) $$\tilde{\lambda }\;:\;=\frac{\nu \lambda +r}{\tilde{\nu }}$$

where $\tilde{\nu }$ are the updated ν, or pseudocounts and $\tilde{\lambda }$ denotes the updated λ, or natural parameters. β is an inverse temperature parameter. In case of Bayes-optimal updates, β=1. ν is referred to as the *pseudocount* as it keeps count of the number of observed data points. $\tilde{\lambda }$ can be interpreted as a weighted mean of the prior λ and the observed data. The responsibilities r, i.e. the accumulated posterior probabilities of observations, can be computed from all messages a factor node F received:

(A18) $$r=\frac{\exp(\log(F(X))+\sum_{m\in ne(F)}^{M}\log(\mu_{v_{m}\to F}(V_{m})))}{\sum_{m\in ne(F)}^{M}\exp(\log(F(X))+\sum_{m\in ne(F)}^{M}\log(\mu_{V_{m_{\to }F}}(V_{m})))}$$

Hypernodes collect responsibilities from all neighbouring factor nodes:

(A19) $$r_{H}\;:\;=\sum_{n\in ne(H)}^{N}r_{n}$$

This way, all observations made at the observable variable nodes in each time step are taken into account. Before message-passing can be instantiated anew, the posterior parameters of all local factor nodes are set to those of their neighbouring hypernode. The exponential family parameterization used in our simulations of healthy vs. chronic pain inference are summarized in Table A1.

**Table A1.** Detailed overview of exponential family parameter settings for the simulation of healthy- and chronic pain inference.

Parameter	Healthy inference	Pathological inference
Prior on 1st node	λ=p(pain)=0.222,	λ=p(pain)=0.777,
	ν=100	ν=100
Top-down prior	$\lambda =p({\text{noxious}}_{t}|{\text{pain}}_{t})=0.8$,	$\lambda =p({\text{noxious}}_{t}|{\text{pain}}_{t})=0.6$,
	$\lambda =p({\text{harmless}}_{t}|{\bar{\text{pain}}}_{t})=0.9$,	$\lambda =p({\text{harmless}}_{t}|{\bar{\text{pain}}}_{t})=0.4$,
	ν=100	ν=20
Transition prior	$\lambda =p(H_{t}=\text{pain}|H_{t-1}=\bar{\text{pain}})=0.2$,	$\lambda =p(H_{t}=\text{pain}|H_{t-1}=\bar{\text{pain}})=0.7$,
	$\lambda =p(H_{t}=\bar{\text{pain}}|H_{t-1}=\text{pain})=0.7$,	$\lambda =p(H_{t}=\bar{\text{pain}}|H_{t-1}=\text{pain})=0.2$,
	ν=100	ν=100
Observations	$S_{1}-S_{5}$= noxious input, $S_{12}-S_{18}$ = harmless input for both cases	

Further, the first observation scheme to produce Figure 2 is shown below. Probabilities and their respective counter-probabilities sum up to 1 (83).

## Appendix B. Derivation of null space and stable pain states

Chronic pain is marked by very tenacious pain perception in the absence of acute tissue damage. This translates to stable pain states in our model. To achieve this stability of hidden states, we need to derive transition probabilities $P({\text{pain}}_{t+1}|{\text{pain}}_{t})$ and $P({\bar{\text{pain}}}_{t+1}|{\bar{\text{pain}}}_{t})$ such that the inferred probability of pain in some next time step t+1 is equal to the probability of pain in the current time step t, which in turn is equal to some stable marginal probability of pain, $P({\text{pain}}_{\infty })$:

(B1) $$P({\text{pain}}_{t+1})=P({\text{pain}}_{t})=P({\text{pain}}_{\infty })$$

The probability of pain in a future time step t+1 is given by the sum over the possible previous states $\in \{\text{pain},\bar{\text{pain}}\}$ and the transition probabilities:

(B2) $$\begin{array}{rl} P({\text{pain}}_{t+1})= & \;P({\text{pain}}_{t+1}|{\text{pain}}_{t})\cdot P({\text{pain}}_{t})\\ & +(1-P({\bar{\text{pain}}}_{t+1}|{\bar{\text{pain}}}_{t})\cdot (1-P({\text{pain}}_{t})) \end{array}$$

Thus, for a stable pain percept after a long time, we require:

(B3) $$\begin{array}{rl} P({\text{pain}}_{\infty })= & \;P({\text{pain}}_{t+1}|{\text{pain}}_{t})\cdot P({\text{pain}}_{\infty })\\ & +(1-P({\bar{\text{pain}}}_{t+1}|{\bar{\text{pain}}}_{t})\cdot (1-P({\text{pain}}_{\infty })) \end{array}$$

Conversely, the condition for a stable no-pain percept is:

(B4) $$\begin{array}{rl} (1-P({\text{pain}}_{\infty }))= & \;(1-P({\text{pain}}_{t+1}|{\text{pain}}_{t}))\cdot P({\text{pain}}_{\infty })\\ & +P({\bar{\text{pain}}}_{t+1}|{\bar{\text{pain}}}_{t})\cdot (1-P({\text{pain}}_{\infty })) \end{array}$$

Solving equation B3 for the transition probabilities, we find

(B5) $$\begin{array}{rl} & P({\text{pain}}_{t+1}|{\text{pain}}_{t})\cdot P({\text{pain}}_{\infty })-P({\bar{\text{pain}}}_{t+1}|{\bar{\text{pain}}}_{t})\cdot (1-P({\text{pain}}_{\infty }))\\ & \;=P({\text{pain}}_{\infty })+P({\text{pain}}_{\infty })-1=2P({\text{pain}}_{\infty })-1 \end{array}$$

Similarly, for $\left(1-P({\text{pain}}_{\infty })\right)$ (Equation B4):

(B6) $$\begin{array}{rl} & -P({\text{pain}}_{t+1}|{\text{pain}}_{t})\;\cdot \;P({\text{pain}}_{\infty })+P({\bar{\text{pain}}}_{t+1}|{\bar{\text{pain}}}_{t})\cdot (1-P({\text{pain}}_{\infty }))\\ & \;=1-P({\text{pain}}_{\infty })-P({\text{pain}}_{\infty })=1-2P({\text{pain}}_{\infty }) \end{array}$$

In matrix-vector form, the last two equations can be written as

(B7) $$\left(\begin{array}{cc} P({\text{pain}}_{\infty }) & -(1-P({\text{pain}}_{\infty }))\\ -P({\text{pain}}_{\infty }) & \left(1-P({\text{pain}}_{\infty })\right) \end{array}\right)\left(\begin{array}{c} P({\text{pain}}_{t+1}|{\text{pain}}_{t})\\ P({\bar{\text{pain}}}_{t+1}|{\bar{\text{pain}}}_{t}) \end{array}\right)=\left(\begin{array}{c} 2P({\text{pain}}_{\infty })-1\\ 1-2P({\text{pain}}_{\infty }) \end{array}\right)$$

To solve for the transition probabilities, we decompose the transition probability vector into the sum of q and r:

(B8) $$(q+r)=\left(\begin{array}{c} P({\text{pain}}_{t+1}|{\text{pain}}_{t})\\ P({\bar{\text{pain}}}_{t+1}|{\bar{\text{pain}}}_{t}) \end{array}\right)$$

where q and r are constrained such that

(B9) $$\left(\begin{array}{cc} P({\text{pain}}_{\infty }) & -(1-P({\text{pain}}_{\infty })\\ -P({\text{pain}}_{\infty }) & \left(1-P({\text{pain}}_{\infty }\right) \end{array}\right)\cdot q=\left(\begin{array}{c} 2P({\text{pain}}_{\infty })-1\\ 1-2P({\text{pain}}_{\infty }) \end{array}\right)$$

in other words, q is that summand of the transition probability decomposition which produces the desired stable marginal probability of pain.

In contrast, for r we require that

(B10) $$\left(\begin{array}{cc} P({\text{pain}}_{\infty }) & -(1-P({\text{pain}}_{\infty }))\\ -P({\text{pain}}_{\infty }) & \left(1-P({\text{pain}}_{\infty }\right) \end{array}\right)\cdot r=0$$

i.e. r is a vector in the null space of the matrix in Equation B7.

Solving for the components of $q=(q_{1},q_{2}{)}^{T}$ and $r=(r_{1},r_{2}{)}^{T}$ yields

(B11) $$\left(\begin{array}{cc} P({\text{pain}}_{\infty }) & -(1-P({\text{pain}}_{\infty })\\ -P({\text{pain}}_{\infty }) & \left(1-P(pain_{\infty }\right) \end{array}\right)\left(\begin{array}{c} q_{1}\\ q_{2} \end{array}\right)=\left(\begin{array}{c} 2P({\text{pain}}_{\infty })-1\\ 1-2P({\text{pain}}_{\infty }). \end{array}\right)$$

i.e.

(B12) $$q_{1}=2P({\text{pain}}_{\infty })-1=-q_{2}$$

Similarly, for r,

(B13) $$\left(\begin{array}{cc} P({\text{pain}}_{\infty }) & -(1-P({\text{pain}}_{\infty }))\\ -P({\text{pain}}_{\infty }) & \left(1-P({\text{pain}}_{\infty })\right) \end{array}\right)\left(\begin{array}{c} r_{1}\\ r_{2} \end{array}\right)=\left(\begin{array}{c} 0\\ 0 \end{array}\right)$$

we find

(B14) $$\frac{P({\text{pain}}_{\infty })}{1-P({\text{pain}}_{\infty })}r_{1}=r_{2}$$

Thus, the transition probabilities for a given $P({\text{pain}}_{\infty })$ can be written as

(B15) $$\left(\begin{array}{c} P({\text{pain}}_{t+1}|{\text{pain}}_{t})\\ P({\bar{\text{pain}}}_{t+1}|{\bar{\text{pain}}}_{t}) \end{array}\right)=\left(\begin{array}{c} 2P({\text{pain}}_{\infty })-1\\ 1-2P({\text{pain}}_{\infty }) \end{array}\right)+\beta \left(\begin{array}{c} 1\\ \frac{P({\text{pain}}_{\infty })}{1-P({\text{pain}}_{\infty })} \end{array}\right)$$

for any β∈R such that $P({\text{pain}}_{t+1}|{\text{pain}}_{t}),$ $P({\bar{\text{pain}}}_{t+1}|{\bar{\text{pain}}}_{t})\in [0,1]$. This implies a one-dimensional null space, where pain percepts remain stable even if the transition probabilities change within this null space (further illustrated in Figure B1). Alternatively, we can eliminate β by solving the first component equation for β and substituting the result into the second component equation:

(B16) $$\begin{array}{rl} P(\bar{{\text{pain}}_{t+1}}|\bar{{\text{pain}}_{t}})= & \frac{1-2P({\text{pain}}_{\infty })}{1-P({\text{pain}}_{\infty })}\\ & +\frac{P({\text{pain}}_{\infty })}{1-P({\text{pain}}_{\infty })}\cdot P({\text{pain}}_{t+1}|{\text{pain}}_{t}) \end{array}$$
